# A study on the therapeutic effect of zero-ray cardiac autonomic ganglion ablation on vasovagal syncope in a special occupational young population

**DOI:** 10.3389/fcvm.2025.1537827

**Published:** 2025-04-03

**Authors:** Yan Guo, Si Li, Xiangyang Yang, Jiaman Hu, Jun Liu, Xiaolong Gu, Yanzhuo Li

**Affiliations:** ^1^ General Hospital of Southern Theater Command, People's Liberation Army, Guangzhou, China; ^2^ Department of Quality Management, Tianjin Rehabilitation Center, Tianjin, China

**Keywords:** zero-ray, anatomical localization method, ablation, high-intensity physical training, young population

## Abstract

**Background:**

This study aims to evaluate the safety and efficacy of zero-ray radiofrequency ablation of the cardiac autonomic ganglionic plexus (GP) for treating vasovagal syncope (VVS) in young individuals undergoing high-intensity physical training.

**Methods:**

We retrospectively analyzed data from 35 young individuals with recurrent syncope (≥3 syncopal episodes within the year prior to the procedure) who underwent GP ablation at our hospital between May 2021 and January 2023. Among them, 33 (94.3%) were male, with a mean age of 22.7 ± 4.6 years. Systemic diseases and/or organic heart conditions that could cause syncope were excluded through comprehensive examinations upon admission. GP ablation was performed in patients with a positive upright tilt test. During the procedure, zero-ray septal puncture was guided by intracardiac ultrasound, and the GP was localized using the anatomical approach (AA) as the ablation target. The ablation endpoint was defined as an increase in heart rate to approximately 90 beats per minute. The safety and efficacy of the procedure were assessed by comparing preoperative and postoperative data, including heart rate, sinus node recovery time, atrioventricular (AV) Wenckebach point, heart rate variability (HRV), deceleration capacity of the heart (DC), and the occurrence of arrhythmias.

**Results:**

No intraoperative or postoperative complications were observed with zero-ray intracavitary ultrasound-guided GP ablation. Postoperatively, the sinus node recovery time and AV Wenckebach point were significantly shorter compared to preoperative values (*P* < 0.001). Both the postoperative mean ECG heart rate and the 12-month postoperative Holter mean heart rate were significantly higher than preoperative levels (*P* < 0.001). Additionally, sDANN-24, rMSSD, and deceleration capacity (DC) were significantly reduced postoperatively (*P* < 0.001). The follow-up period ranged from a minimum of 15 months to a maximum of 35 months. Within one year after surgery, two cases experienced a single episode of syncope, and one case reported a single episode of a syncopal premonitory aura. In the patient with a syncopal premonitory aura, outpatient ECG and Holter monitoring showed no abnormalities. The patient who experienced syncope was readmitted for further evaluation, including ECG, Holter monitoring, and an upright tilt test, which was negative. Two postoperative cases (one with a syncopal premonitory aura and one without syncope) exhibited second-degree type II AV block on Holter monitoring, which occurred during nocturnal sleep. Despite this, both groups were able to continue high-intensity physical training with significant symptomatic improvement.

**Conclusions:**

Zero-ray cardiac GP ablation is a radiation-free, minimally invasive, safe, and effective treatment for young VVS patients undergoing high-intensity physical training.

## Introduction

It is well established that dysregulation of the autonomic nervous system (ANS) is the primary etiology of vasovagal syncope (VVS) (1). Young individuals undergoing high-intensity physical training represent a unique population prone to sympathetic excitation, increased heart rate, and peripheral vasodilation, leading to blood redistribution, forceful ventricular contraction, and an “emptying effect.” These physiological changes can reflexively trigger heightened vagal excitability in specific environments or activities. Due to abnormal vagal regulation, this population is not only more susceptible to VVS characterized by a sudden drop in blood pressure and/or heart rate resulting in insufficient cerebral perfusion but is also at an increased risk of trauma associated with syncopal episodes. Currently, most VVS patients are managed conservatively through pharmacological therapy and lifestyle modifications. However, medications often have significant side effects and limited efficacy, while lifestyle interventions, such as tilt training, are poorly adhered to and yield suboptimal outcomes (2–4). Pacing therapy, though an option, is an extreme measure that can negatively impact both occupational and mental well-being, making it difficult for patients and their families to accept. Therefore, identifying a more effective approach to achieve long-term inhibition of vagal reflexes is particularly critical for this population. Since 2005, Pachon et al. have utilized GP ablation to treat functional atrioventricular (AV) block and sinus node dysfunction (5). Although this technique has evolved from an experimental procedure to being implemented in numerous major hospitals worldwide (6–9), the number of patients undergoing surgery remains limited, particularly in this specific population. Moreover, studies focusing on individuals engaged in high-intensity physical training are currently lacking. The human heart contains at least six known cardiac ganglionated plexuses (GPs), with four major left atrial GPs located around the pulmonary venous sinus (10). In this study, ablation was performed on the four primary left atrial GPs: the left superior GP (LSGP), left inferior GP (LIGP), right superior GP (RSGP), and right inferior GP (RIGP), in that order.

## Methods

### Study population

This study included 35 young individuals undergoing high-intensity physical training who were admitted to our hospital for recurrent syncope, diagnosed with vasovagal syncope (VVS), and underwent GP ablation between May 2021 and January 2023. All patients experienced frequent syncopal episodes (≥3 episodes within one year), which significantly impacted their physical training and daily life. According to the 2019 ACC/AHA/HRS guidelines for the evaluation and management of syncope and the 2018 ESC guidelines for the diagnosis and management of syncope, comprehensive laboratory tests and examinations were performed upon admission. Routine evaluations included skull MRI, electroencephalography (EEG), and laboratory tests to exclude alternative causes of syncope, such as epilepsy, stroke, cerebral artery stenosis, hyperlipidemia, anemia, infection, malnutrition, hyperthyroidism, diabetes, and trace element deficiencies. None of the patients had risk factors associated with heart disease, such as advanced age or menopause, nor did they have a history of smoking or alcohol consumption. To rule out structural and functional cardiac abnormalities, all patients underwent electrocardiography (ECG), echocardiography, treadmill exercise testing, Holter monitoring, blood pressure monitoring, and electrophysiological studies (EPS). Additional assessments, including myocardial infarction markers and B-type natriuretic peptide (BNP) levels, were conducted to exclude conditions such as hypertension, cardiomyopathy, coronary artery disease, valvular heart disease, and pathological atrioventricular block. General patient characteristics are summarized in Table 1.

**Table 1:** Preoperative basic patient information data (*n* = 35).

Patients	Follow-up 25.03 ± 5.91 months (15–35 months)
Age (years)	22.7 ± 4.6
Male [*n* (%)]	33/35 (94.3%)
Number of episodes in the 1 year before surgery [*n* (%)]	3.43 ± 1.09
Activity-related [*n* (%)]	29/35 (83%)
Left ventricular ejection fraction LVEF (%)	64.6 ± 5.8
Left atrial anteroposterior diameter LAd (mm)	28.3 ± 2.8
Left atrial transverse diameter LAs(mm)	33.1 ± 3.5
Left atrial upper and lower diameter LAI(mm)	44.5 ± 4.5
Hemoglobin (g/L)	144.3 ± 12.0
Total magnesium (mmol/24 h)	0.9 ± 0.1
Total sodium (mmol/24 h)	140.9 ± 2.0
Total potassium (mmol/24 h)	4.1 ± 0.3
Urine specific gravity	1.0 ± 0.0
Cardioinhibitory [*n* (%)]	9/35 (25.7%)
Vasopressor type [*n* (%)]	1/35 (2.9%)
Mixed type [*n* (%)]	25/35 (71.4%)

**Figure 1:**
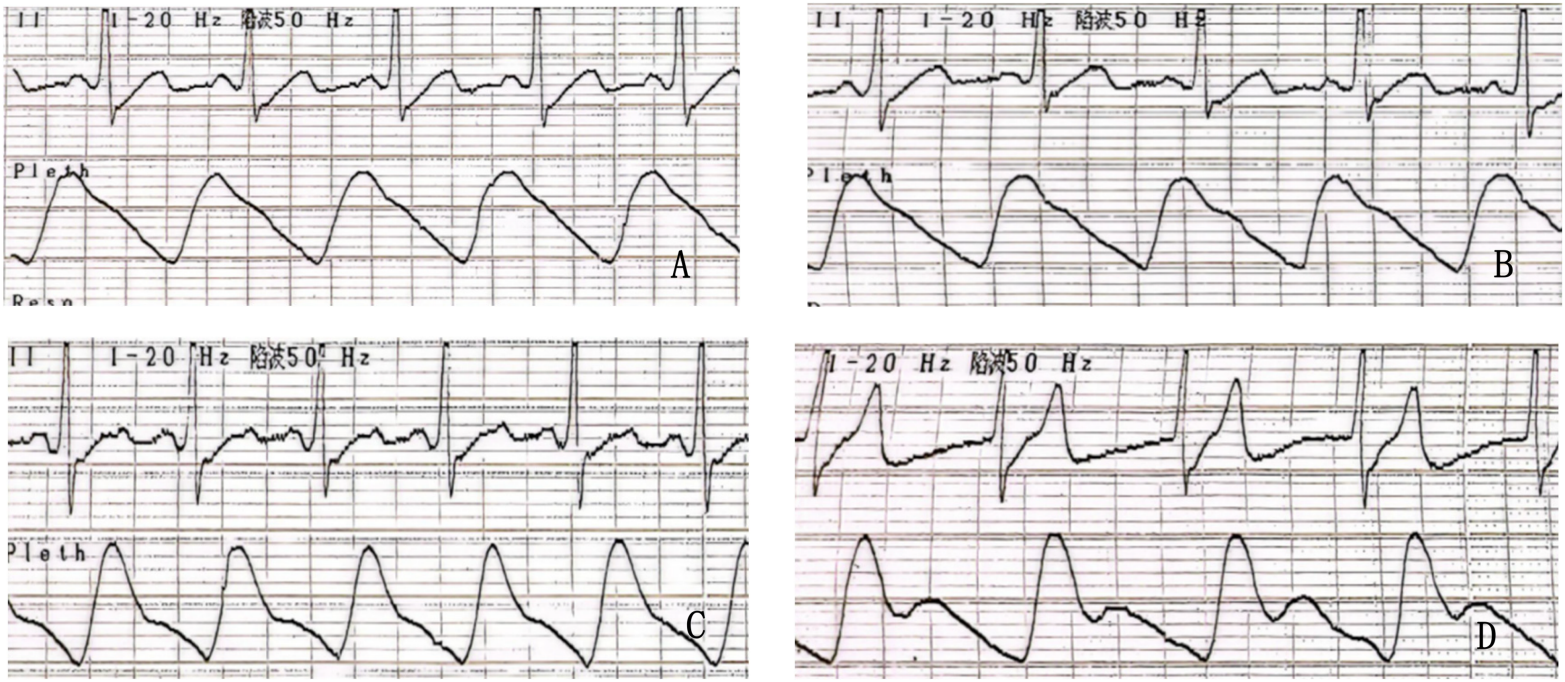
Upright tilt test showing a positiv (mixed) result before ablation: syncopal episode with junctional fugitive rhythm, slowed heart rate, and decreased blood pressure (pre-episode blood pressure: 120/65 mmHg, episode blood pressure: 85/40 mmHg) on ECG. **(A)** ECG at 0° (lying position), **(B)** ECG at 70° (tilting), **(C)** ECG after administration of sublingual nitroglycerin (0.375 mg), showing an increased heart rate compared to the lying and tilting positions. **(D)** ECG during the positive episode.

Physical stress training and β-blocker therapy were either ineffective or poorly tolerated in this population, leading to the decision to perform cardiac GP radiofrequency ablation. All patients underwent an upright tilt test, with positive results confirming VVS. Based on the Vasovagal Syncope International Study classification, positive responses were categorized into mixed, cardioinhibitory, and vasodepressor types (11–13), as shown in Figure 1.

### Study methodology

**Figure 2:**
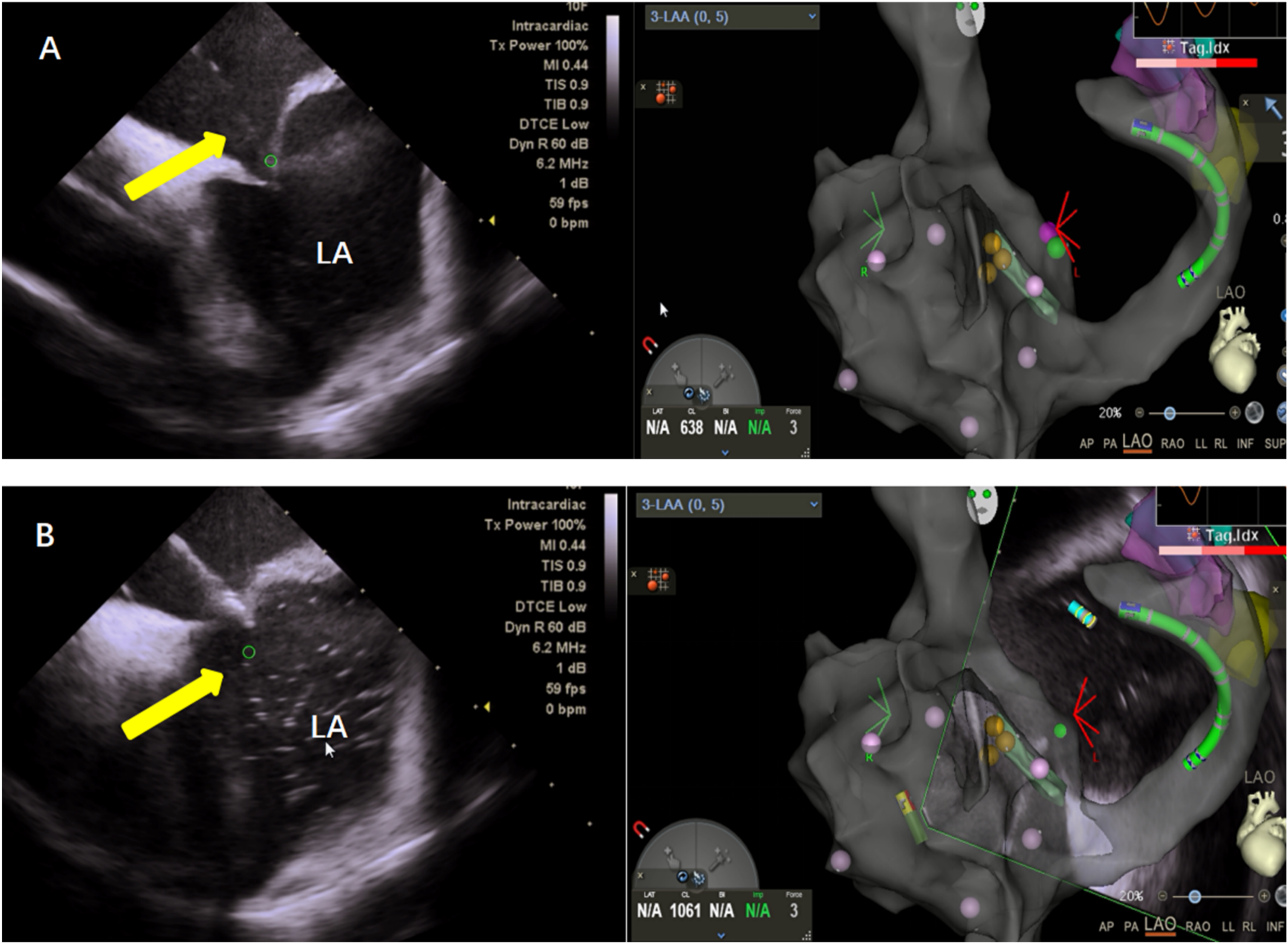
**(A)** Zero ray puncture: intracardiac ultrasound (left), showing puncture of the atrial septum under the guidance of the right atrial model and three-dimensional visualization of a patent foramen ovale (right). **(B)** Saline was injected into the puncture needle under direct visualization with intracardiac ultrasound (left), confirming successful puncture of the atrial septum.

**Figure 3:**
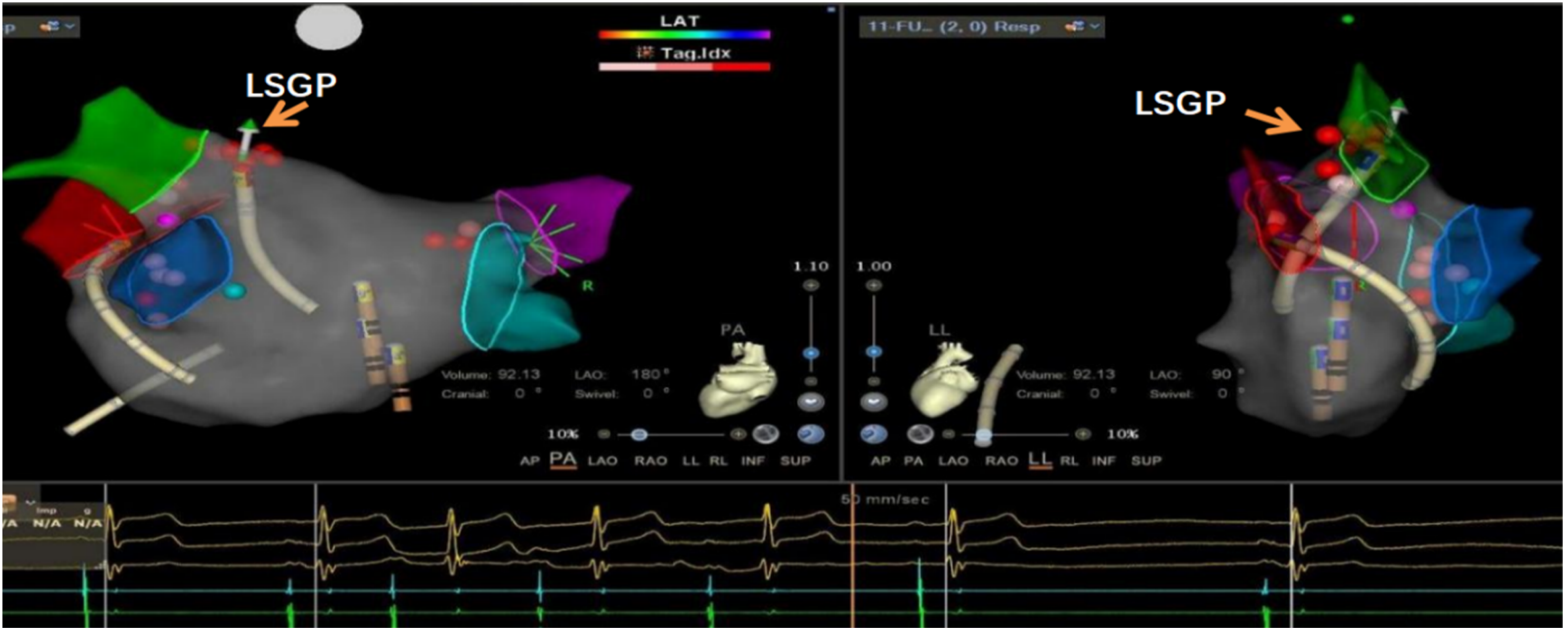
Significant sinus bradycardia and first-degree atrioventricular block after ablation of the left upper ganglionated plexus (LSGP) of the left atrium, indicating that this was the target ablation site.

**Figure 4:**
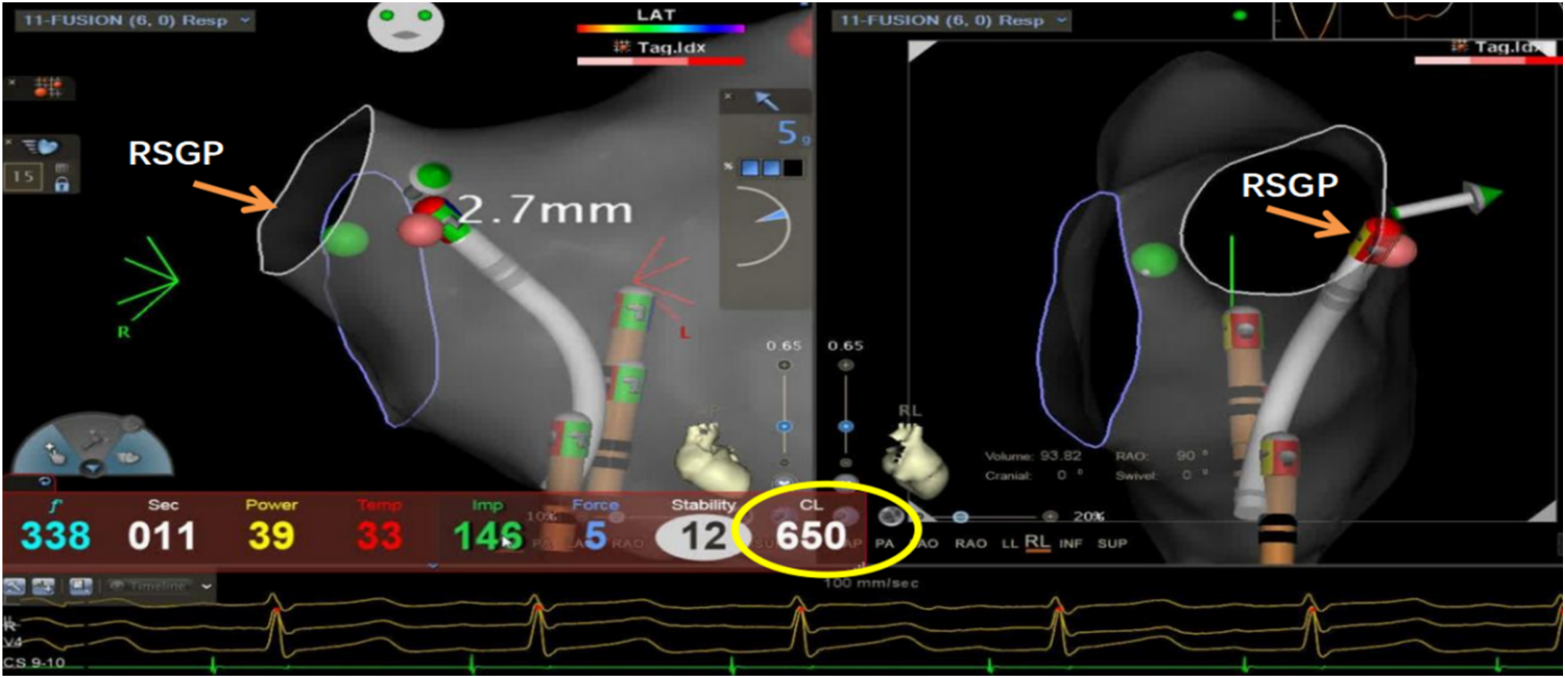
Ablation of the left atrial right-superior ganglionated plexus (RSGP) resulted in a gradual increase in sinus heart rate to approximately 92 beats per minute (RR interval 650 ms), after which the procedure was terminated.

**Figure 5:**
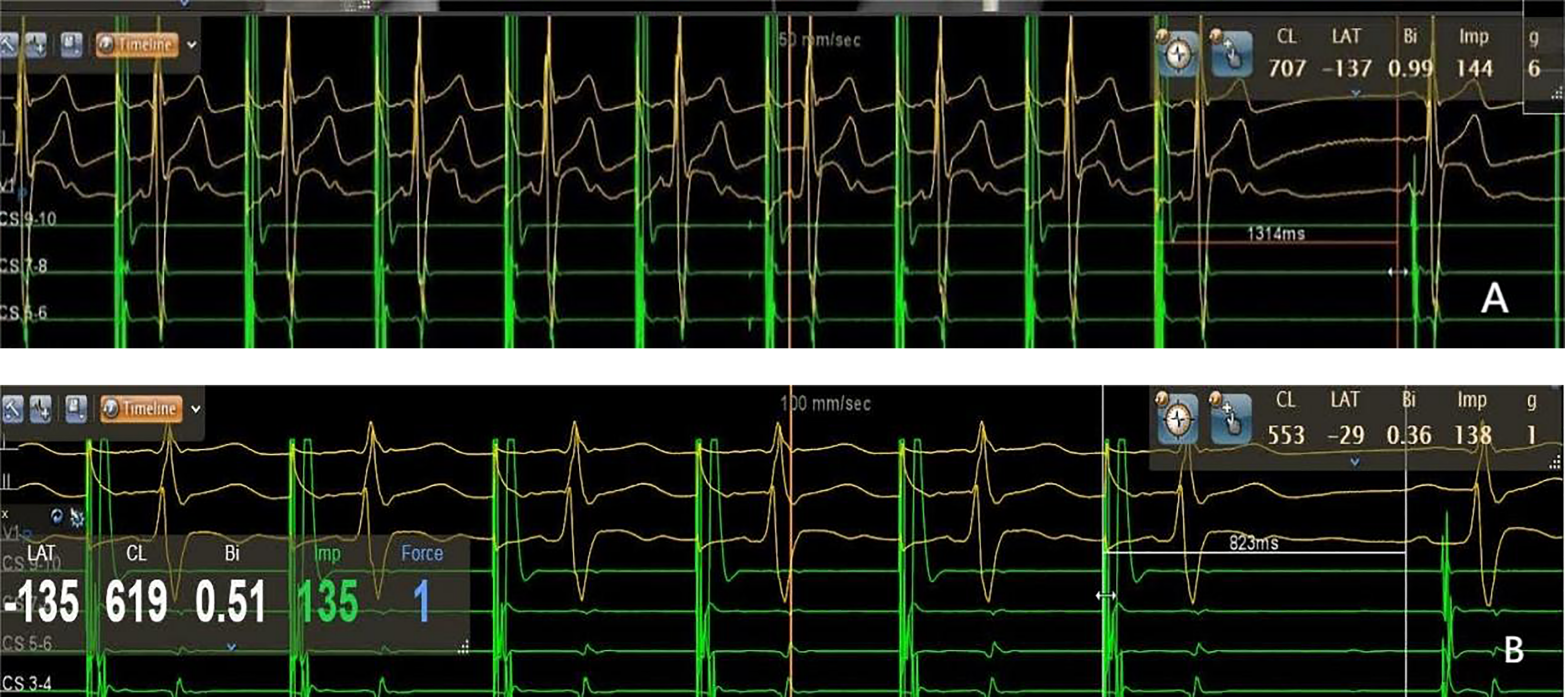
Sinus node recovery time measured by intracardiac electrophysiological examination. A: 1,314 ms pre-procedure, B: 823 ms post-procedure. The sinus node recovery time was significantly shortened post-ablation (*p* < 0.001).

This study utilized the Carto electrophysiological navigation system (CARTO3, FG-5400-00C, USA) for cardiac modeling and ablation. Under intracardiac ultrasound guidance, a right atrial model was established, and three-dimensional fossa ovalis positioning was performed. Zero-ray transseptal puncture was carried out, and the SL1 sheath was advanced into the left atrium, as shown in Figure 2. Using the left atrial model for guidance, GP ablation was performed based on anatomical localization (temperature-controlled at 43°C, 40-watt discharge, with cold saline irrigation). During GP ablation, vagal responses including significant sinus bradycardia, sinus arrest, and atrioventricular block were observed, as shown in Figure 3. Ablation sites were marked, and ablated around these points reinforcely until vagal responses were no longer elicited. GP ablation was sequentially performed in the left superior GP (LSGP), left inferior GP (LIGP), right superior GP (RSGP), and right inferior GP (RIGP), as illustrated in Figure 2B. If five consecutive ablations within a GP region failed to induce any vagal response, the procedure proceeded to the next GP region. In most cases (80%), if the sinus rhythm gradually increased to approximately 90 beats per minute during ablation of the right superior pulmonary vein GP, ablation in that area was terminated at that point, as shown in Figure 4. The increase in sinus rhythm, disappearance of vagal reflexes, and completion of anatomical ablation signified the end of the procedure. Electrophysiological studies (EPS) demonstrated that the sinoatrial node recovery time and AV Wenckebach point were significantly shortened postoperatively compared to preoperative values, as shown in Figure 5.

### Holter detection of arrhythmia, HRV and DC analysis

Using the Holter system (CB-1306-C, Wuxi Zhongjian Science Instrument Co., Ltd.), 24-hour ECG data of the patient was recorded, and heart rate variability (HRV) and dynamic correlation (DC) were analyzed using both computer software and manual methods. HRV analysis involved excluding abnormal QRS waveforms, after which the software calculated the standard deviation of the 24-hour NN intervals of sinus rhythm QRS waveforms (SDNN-24) and the root mean square of successive differences (rMSSD). DC was calculated using a bit-phase whole-sequence signal averaging algorithm. Atrial and ventricular premature beats were excluded to avoid errors from artifacts, retaining only R-R intervals. Heartbeat intervals longer than the previous interval were defined as anchor points. The 15 R-R intervals before and after the anchor point were selected as fixed heart rate segments and aligned at the anchor point. The signal X within these aligned segments was then averaged. Here, X0 represents the average of all R-R interval signals at the center of the heart rate segment, X1 is the average of the first R-R interval after X0, and X-1 and X-2 are the averages of the two R-R intervals before X0. DC was quantified using the formula: DC = 1/4 (X0 + X1 - X-1 - X-2) (14, 15).

### Patient follow-up

All patients took aspirin for 1 month. ECG was rechecked on the day of ablation, and follow-up calls were made at 1, 3, 6, and 12 months post-operation to inquire about the patients' blood pressure, heart rate, and any recurrence of syncope or pre-syncope symptoms, including dizziness, fatigue, or palpitations. If syncope precursors were reported, patients were advised to undergo an ECG and Holter test at the outpatient clinic. If necessary, an upright tilt test was recommended at the hospital. In the event of a new syncopal episode, readmission for evaluation and repeat tilt test was recommended, and if positive,a second GP ablation was offered. The Holter test was reviewed in the outpatient clinic 12 months after surgery. For patients unable to visit the hospital, phone contact was made to collect Holter results from their local hospital for final analysis.

### Statistical analysis

Data were analyzed using SPSS 26.0. Continuous variables that followed a normal distribution were expressed as mean ± standard deviation (x ± s), while data not meeting normal distribution were described using the median and interquartile range. For normally distributed samples, the independent two-sample t-test was used, and for non-normally distributed samples, the Mann–Whitney *U*-test was applied. Categorical variables were expressed as percentages and analyzed using the chi-square test or the corrected chi-square test. Differences were considered statistically significant at *p* < 0.05.

## Results of the study

**Figure 6:**
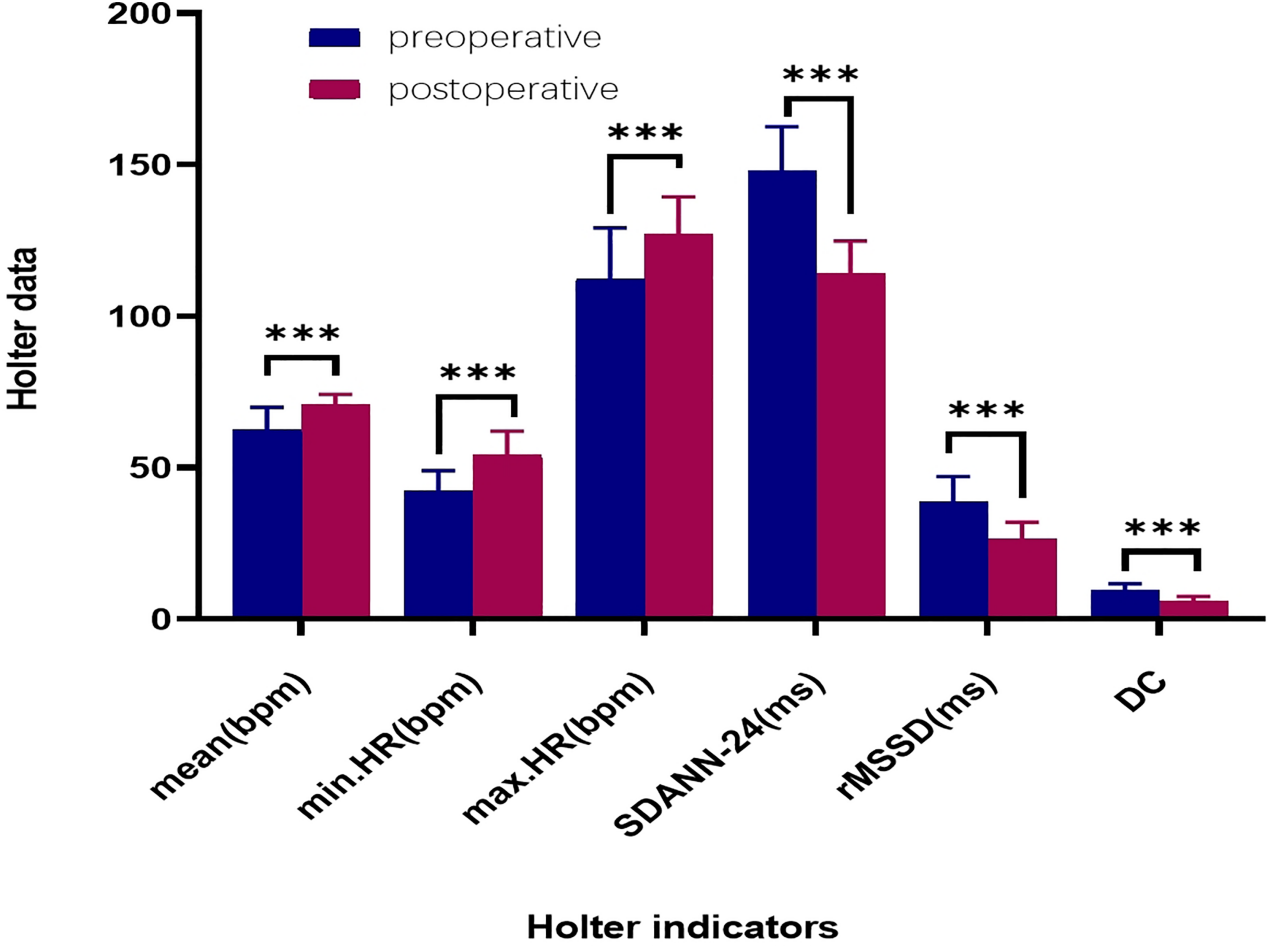
Comparison of preoperative and 12-month postoperative holter data. ****: *p* < 0.001. HR: heart rate (bpm); mean.HR: mean heart rate; max.HR: maximum heart rate; min.HR: minimum heart rate; SDANN-24: standard deviation of the 24-hour NN interval; RMSSD: root mean square of successive differences; DC: deceleration capacity of the heart.

**Figure 7:**
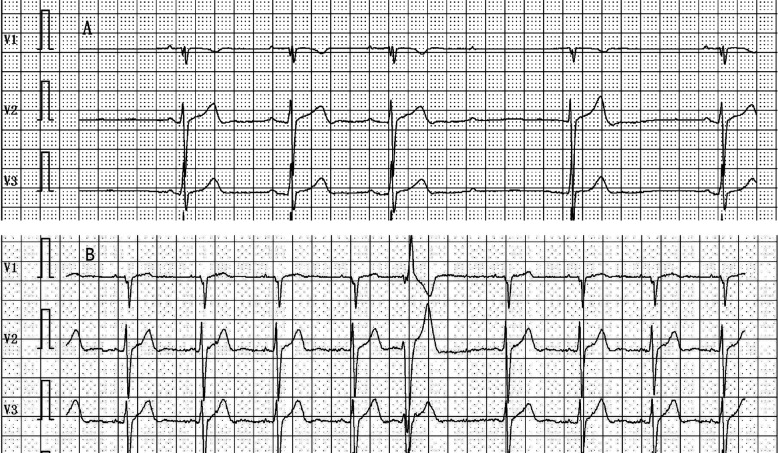
**(A)** Holter monitoring recorded the occurrence of slow-type arrhythmias, including second-degree atrioventricular block during nighttime sleep, pre-procedure. **(B)** Holter monitoring recorded no significant sinus bradycardia, junctional escape, or slow-type arrhythmias (including second-degree atrioventricular block) post-procedure.

All 35 patients underwent the AA procedure, with a mean total operative time of 111.43 ± 9.22 min, including disinfection, draping, and hemostatic dressing. The postoperative AV Wenckebach point was significantly shorter [440.0 [400.0, 500.0] ms vs. 360.0 [340.0, 380.0] ms, *P* < 0.001]. Additionally, the postoperative sinus node recovery time was significantly shorter [1,287.0 [1,214.0, 1,356.0] ms vs. 865.0 [823.0, 894.0] ms, *P* < 0.001]. The ECG heart rate on the postoperative day was significantly faster than preoperatively [90.0 [78.0, 97.0] vs. 59.0 [54.0, 69.0] beats/min, *P* < 0.001]. There were no intraoperative or postoperative complications, as shown in Table 2. At the 12-month follow-up, Holter monitoring showed that the mean postoperative heart rate was faster than preoperative values (*P* < 0.001). Both the slowest and fastest heart rates were significantly increased (*P* < 0.05). HRV and DC analysis using Holter data and specific software revealed that postoperative SDANN-24 (ms) and rMSSD (ms) were significantly shorter compared to preoperative values (*P* < 0.001), and postoperative DC was lower than preoperative (9.75 ± 1.98 vs. 6.1 ± 1.3, *P* < 0.001), as shown in Figure 6. The incidence of Holter-detected arrhythmias (including sinus bradycardia, junctional escape, and slow-type arrhythmias of degree II and above) was 45.7% preoperatively and 5.7% postoperatively, with a statistically significant difference (*P* < 0.001), as shown in Figure 7. In the 12 months following surgery, there were 2 cases of syncope (one episode each) and 1 case of syncopal precursor (one episode) reported. The 2 syncope cases were readmitted for review, with normal ECG and Holter results and a negative upright tilt test. The case with syncopal precursor had a normal ECG and Holter result during the outpatient review. All patients showed significant symptom improvement and effective surgical outcomes, as shown in Table 3.

**Table 2:** Comparison of electrocardiographic parameters before and after cardiac autonomic ganglion plexus ablation.

Program	Preoperative	Postoperative	*P*
AV Wenckebach piont (ms)	440.0 (400.0,500.0)	360.0 (340.0,380.0)	<0.001
Sinus node recovery time (ms)	1,287.0 (1,214.0,1,356.0)	865.0 (823.0,894.0)	<0.001
ECG heart rate (beats/min)	59.0 (54.0,69.0)	90.0 (78.0,97.0)	<0.001

**Table 3:** Comparison of outcomes in the long-term period after cardiac autonomic ganglion plexus ablation.

Program	Preoperative	Postoperative	*P*
Fainting or presyncope [*n* (%)]	35/35 (100%)	3/35 (8.6%)	<0.001
Physical training limitations [*n* (%)]	29/35 (82.9%)	0/35 (0%)	<0.001
Positive upright tilt test [*n* (%)]	35/35 (100%)	0/35 (0%)	<0.001
Trauma due to syncope [*n* (%)]	28/35 (80%)	0/35 (0%)	<0.001
Arrhythmia [*n* (%)]	16/35 (45.7%)	2/35 (5.7%)	<0.001

## Discussion

Syncope is a transient loss of consciousness caused by overall cerebral underperfusion, characterized by brief, spontaneous recovery (12). Etiological investigations have revealed that vasovagal reflexes or neurocardiogenic reflexes are the most common causes of syncope (16). Although vasovagal syncope (VVS) has not been shown to affect morbidity and mortality directly, sudden falls resulting from syncope often lead to injuries (17–19). In this study, up to 80% of patients experienced varying degrees of trauma related to VVS, highlighting the importance of addressing VVS in the young population engaged in high-intensity or specialized physical training. GP ablation, an emerging therapeutic approach for VVS, has been reported both domestically and internationally. However, due to stringent and complex inclusion criteria, high demands for surgical technique and equipment, and the varying impact of symptoms on patients’ lives and work, the number of individuals undergoing the procedure remains limited. Furthermore, no studies have yet been reported on this procedure in young individuals involved in specialized occupations.

In this study, we analyzed the safety and efficacy of GP ablation by reviewing the data of young individuals who had previously undergone GP ablation while engaged in high-intensity physical training. We compared preoperative and postoperative clinical data. Drawing on the experience of Yao Yan, Rivarola, Pachon, and others who have reported on VVS treatment (14, 20–22), GP ablation was performed on 35 patients using the zero-ray AA procedure. While a small portion of individuals underwent RIGP ablation, the majority did not for two main reasons: first, there were fewer excitatory points in the vagus nerve segment of the right ganglion plexus (RIGP); second, during ablation of the RSGP, most patients experienced an increase in heart rate and disappearance of the vagus nerve reflex. Performing RIGP ablation next could potentially lead to adverse reactions, such as sinus tachycardia caused by the procedure. The entire procedure was radiation-free, with an average time of less than two hours, and no intraoperative or postoperative surgical complications occurred. Postoperative AV Wenckebach point and sinus node recovery time were both shorter than preoperative values, suggesting a reduction in postoperative vagal excitability and an increase in sympathetic activity. On the day of surgery, the heart rate was significantly higher than before, and the average heart rate on Holter monitoring 12 months after surgery was lower than on the day of surgery but still higher than preoperative levels (*P* < 0.001). This indicates that vagal inhibition was most pronounced following the procedure immediately, with reduced excitability of vagus nerve, though a little recovery over during the observation period. Heart rate variability (HRV) is used to assess cardiac autonomic nerve activity, reflecting the balance between sympathetic and parasympathetic control. Holter recordings are considered the “gold standard” for clinical HRV assessment (23). Postoperative SDANN-24 (ms) and rMSSD (ms), key HRV indicators, were significantly shorter than preoperative values (*P* < 0.001), which aligns with previous studies (24–27). Additionally, some studies have concluded that HRV is an important predictor of negative outcomes (28). To further assess cardiac autonomic function, DC was introduced to quantitatively evaluate cardiac vagal function. The reduction of cardiac DC reflects a decrease in vagal tone, and this finding is consistent with the relevant literature (15). The results of the present study align with previous literature, showing that postoperative DC was reduced compared to preoperative DC. Preoperative Holter recordings from some patients revealed sinus bradycardia, junctional escape, and slow-type arrhythmias of degree II and above during nighttime sleep, which were attributed to vagal nerve activity in the heart. After surgery, this phenomenon was significantly reduced (*P* < 0.001). Within 12 months post-surgery, there were two cases of syncope recurrence and one case of syncope precursor. Analysis showed that the total number of GP ablations in these three patients was three, and the surgery duration was shorter than the average. This suggests that the anatomical method or a shorter ablation time may have played a role in the results. It cannot be ruled out that some ganglion plexuses, such as RIGP,obtuse marginal (near VoM)and inferior paraseptal (along CS) plexi, may have been overlooked. However, the number of postoperative episodes significantly decreased, and relevant examination results were negative, indicating that the overall treatment was effective.

In this study, with a minimum follow-up of 15 months and a maximum follow-up of 35 months, all patients continued to perform high-intensity physical training without any physical impairments related to syncope. Additionally, there was a significant reduction in the number of prodromal symptoms per year after autonomic denervation. Unlike some studies that report recurrence rates with GP ablation (14, 29), this population showed a low recurrence rate, did not require a second procedure, and experienced no surgical complications (0%) with faster postoperative recovery. This may be attributed to the study population's young age and predominantly male composition, which likely led to unique neuromodulation characteristics or the effects of long-term high-intensity physical training on neural excitability. As a result, the procedure proved to be effective for cardiac GP treatment, and the zero-irradiation approach was beneficial both for patients and healthcare professionals.

According to available data, cardiac GP ablation offers an alternative treatment for young VVS patients who urgently need to return to specialized professions or training. It helps meet the demands of their unique environments, roles, and high-intensity physical activity requirements, improving the quality of life for this population while providing an effective solution for safe training and work. Additionally, it eliminates the need for pacemaker implantation in patients with severe conduction block caused by vagal hypertonia, significantly reducing both psychological burdens and family financial costs.

## Study limitations

This is a single-center, early series involving a small number of high-intensity physical training young VVS patients, 94.29% of whom were male due to occupational specificity, with mixed types accounting for 71.4% of the entire study population. A larger cohort is needed to confirm the safety and efficacy of this new treatment option for this population. Additionally, randomized controlled trials comparing this approach to general populations and more VVS with cardioinhibitory may be required to further establish the superiority of this method. Furthermore, this preliminary study did not compare specific surgical parameters based on upright tilt test results, nor did it analyze the number and duration of ablations at each GP point during surgery. some ganglionic plexuses may have been overlooked, potentially affecting both surgical time and effectiveness. In short follow up, we have shown that zero-ray GP ablation is effective in preventing and treating frequent VVS episodes in young patients undergoing intense physical training, though long-term follow-up data for this procedure are still lacking. Due to the specific nature of the patients’ occupations, implantable loop recorder (ILR) testing could not be performed. If this technology were available to record heart rhythm onset, the results could be more comprehensive. Despite these limitations, our findings offer a novel approach to managing frequent VVS in this specialized occupational group, contributing valuable insights to the existing literature.

## Data Availability

The datasets presented in this article are not readily available because due to the special identity of the patients, we have signed a confidentiality agreement for the original data. Requests to access the datasets should be directed to Yan Guo, 1013155928@qq.com.

## References

[1] Aksu T Davila A Gupta D. The “heart brain” and neuromodulation for vasovagal syncope. Auton Neurosci. (2021) 12:236. 10.1016/j.autneu.2021.102892 34666205

[2] Gurevitz O Barsheshet A Bar-Lev D Zimlichman E Rosenfeld GF Benderly M Tilt training: does it have a role in preventing vasovagal syncope? Pacing Clin Electrophysiol. (2007) 30:1499–505. 10.1111/j.1540-8159.2007.00898.x 18070305

[3] Hatoum T Raj S Sheldon RS. Current approach to the treatment of vasovagal syncope in adults. Intern Emerg Med. (2023) 18(1):23–30. 10.1007/s11739-022-03102-w 36117230

[4] Duygu H Zoghi M Turk U Akyuz S Ozerkan F Akilli A The role of tilt training in preventing recurrent syncope in patients with vasovagal syncope: a prospective and randomized study. Pacing Clin Electrophysiol. (2008) 31:592. 10.1111/j.1540-8159.2008.01046.x 18439174

[5] Pachon JC Pachon EI Pachon JC Lobo TJ Pachon MZ Vargas RNA “Cardioneuroablation”–new treatment for neurocardiogenic syncope,functional AV block and sinus dysfunction using catheter RF-ablation. Europace. (2005) 7(1):1–13. 10.1016/j.eupc.2004.10.003 15670960

[6] Aksu T Golcuk E Yalin K Guler TE Erden I. Simplified cardioneuroablation in the treatment of reflex syncope, functional AV block, and sinus node dysfunction. Pacing Clin Electrophysiol. (2016) 39(1):42–53. 10.1111/pace.12756 26411271

[7] Qin M Zhang Y Liu X Jiang W-F Wu S-H Po S. Atrial ganglionated plexus modification: a novel approach to treat symptomatic sinus bradycardia. JACC Clin Electrophysiol. (2017) 3(9):950–9. 10.1016/j.jacep.2017.01.022 29759719

[8] Aksu T Guler TE Mutluer FO Bozyel S Golcuk SE Yalin K. Electroanatomic-mapping-guided cardioneuroablation versus combined approach for vasovagal syncope: a cross-sectional observational study. J Interv Card Electrophysiol. (2019) 54(2):177–88. 10.1007/s10840-018-0421-4 30054828

[9] Hu F Zheng L Liang E Ding L Wu L Chen G Right anterior ganglionated plexus: the primary target of cardioneuroablation? Heart Rhythm. (2019) 16(10):1545–51. 10.1016/j.hrthm.2019.07.018 31330187

[10] Aksu T Guler TE Bozyel S Yalin K. Vagal responses during cardioneuroablation on different ganglionated plexi: is there any role of ablation strategy? Int J Cardiol. (2020) 304:50–5. 10.1016/j.ijcard.2019.12.003 31836362

[11] Sutton R Fedorowski A Olshansky B van Dijk JG Abe H Brignole M Affiliations expand. Tilt testing remains a valuable asset. Eur. Heart J. (2021) 42(17):1654–60. 10.1093/eurheartj/ehab084 33624801 PMC8245144

[12] Goldberger ZD Petek BJ Brignole M Shen W-K Sheldon RS Solbiati M ACC/AHA/HRS versus ESC guidelines for the diagnosis and management of syncope: JACC guideline comparison. J Am Coll Cardiol. (2019) 74(19):2410–23. 10.1016/j.jacc.2019.09.012 31699282

[13] Cooper PN Sutton R. Tilt testing. Pract Neurol. (2023) 23(6):493–500. 10.1136/pn-2023-003749 37726165

[14] Yao Y Shi R Wong T Zheng L Chen W Yang L Endocardial autonomic denervation of the left atrium to treat vasovagal syncope: an early experience in humans. Circ Arrhythm Electrophysiol. (2012) 5(2):279–86. 10.1161/CIRCEP.111.966465 22275485

[15] Zheng L Sun W Liu S Liang E Du Z Guo J The diagnostic value of cardiac deceleration capacity in vasovagal syncope. Circ Arrhythm Electrophysiol. (2020) 13(12):e008659. 10.1161/CIRCEP.120.008659 33197331

[16] Fedorowski A Kulakowski P Brignole M de Lange FJ Kenny RA Moya A Twenty-five years of research on syncope. Europace. (2023) 25(8):euad163. 10.1093/europace/euad163 37622579 PMC10450792

[17] Jorge JG Pournazari P Raj SR Maxey C Sheldon RS. Frequency of injuries associated with syncope in the prevention of syncope trials. Europace. (2020) 22:1896–903. 10.1093/europace/euaa246 32954415 PMC7758472

[18] Jorge JG Raj SR Teixeira PS Teixeira JA Sheldon RS. Likelihood of injury due to vasovagal syncope: a systematic review and meta-analysis. Europace. (2021) 23:1092–9. 10.1093/europace/euab041 33693816

[19] Soteriades ES Evans JC Larson MG Chen MH Chen L Benjamin EJ Incidence and prognosis of syncope. N Engl J Med. (2002) 347(12):878–85. 10.1056/NEJMoa012407 12239256

[20] Rivarola EW Hachul D Wu T Pisani C Hardy C Raimundi F Targets and end points in cardiac autonomic denervation procedures. Circ Arrhythm Electrophysiol. (2017) 10(2):e004638. 10.1161/CIRCEP.116.004638 28202630

[21] Pachon MJC Pachon MEI Pachon MJC Lobo TJ Pachon MZ Vargas RN A new treatment for atrial fibrillation based on spectral analysis to guide the catheter RF-ablation. Europace. (2004) 6(6):590–601. 10.1016/j.eupc.2004.08.005 15519263

[22] Rivarola E Hardy C Sosa E Hachul D Furlan V Raimundi F Selective atrial vagal denervation guided by spectral mapping to treat advanced atrioventricular block. Europace. (2016) 18(3):445–9. 10.1093/europace/euv142 26071235

[23] Lombardi F Stein PK. Origin of heart rate variability and turbulence: an appraisal of autonomic modulation of cardiovascular function. Front Physiol. (2011) 2:95. 10.3389/fphys.2011.00095 22163222 PMC3233900

[24] Brignole M Aksu T Calò L Debruyne P Deharo JC Fanciulli A Clinical controversy: methodology and indications of cardioneuroablation for reflex syncope. Europace. (2023) 25(5):euad033. 10.1093/europace/euad033 37021351 PMC10227654

[25] Soni B Gupta D Gopinathannair R. Quality of life improvement following cardioneuroablation for vasovagal syncope: expected or too early to say? J Interv Card Electrophysiol. (2023). 10.1007/s10840-023-01489-w 36705871

[26] Piotrowski R Baran J Sikorska A Krynski T Kulakowski P. Cardioneuroablation for reflex syncope: efficacy and effects on autonomic cardiac regulation—a prospective randomized trial. JACC Clin Electrophysiol. (2023) 9(1):85–95. 10.1016/j.jacep.2022.08.011 36114133

[27] Pachon JCM Pachon EIM Pachon MZC Lobo TJ Pachon JCM Santillana TGP. Catheter ablation of severe neurally meditated reflex (neurocardiogenic or vasovagal) syncope: cardioneuroablation long-term results. Europace. (2011) 13(9):1231–42. 10.1093/europace/eur163 21712276

[28] Onishi Y Minoura Y Chiba Y Onuki T Ito H Adachi T Daily dysfunction of autonomic regulation based on ambulatory blood pressure monitoring in patients with neurally mediated reflex syncope. Pacing Clin Electrophysiol. (2015) 38:997–1004. 10.1111/pace.12661 25974151

[29] Pachon-M JC Pachon-M EI Pachon CTC Santillana-P TG Lobo TJ Pachon-M JC Long-Term evaluation of the vagal denervation by cardioneuroablation using holter and heart rate variability. Circ Arrhythm Electrophysiol. (2020) 13(12):e008703. 10.1161/CIRCEP.120.008703 33198486

